# DS-MENet for the classification of citrus disease

**DOI:** 10.3389/fpls.2022.884464

**Published:** 2022-07-22

**Authors:** Xuyao Liu, Yaowen Hu, Guoxiong Zhou, Weiwei Cai, Mingfang He, Jialei Zhan, Yahui Hu, Liujun Li

**Affiliations:** ^1^College of Computer & Information Engineering, Central South University of Forestry and Technology, Changsha, China; ^2^Plant Protection Research Institute, Hunan Academy of Agricultural Sciences, Changsha, China; ^3^Department of Civil, Architectural and Environmental Engineering, University of Missouri-Rolla, Rolla, MO, United States

**Keywords:** citrus disease detection, depthwise separable convolution, ReMish, multi-channel fusion backbone enhancement method, DS-MENet, image enhancement

## Abstract

Affected by various environmental factors, citrus will frequently suffer from diseases during the growth process, which has brought huge obstacles to the development of agriculture. This paper proposes a new method for identifying and classifying citrus diseases. Firstly, this paper designs an image enhancement method based on the MSRCR algorithm and homomorphic filtering algorithm optimized by Laplacian (HFLF-MS) to highlight the disease characteristics of citrus. Secondly, we designed a new neural network DS-MENet based on the DenseNet-121 backbone structure. In DS-MENet, the regular convolution in Dense Block is replaced with depthwise separable convolution, which reduces the network parameters. The ReMish activation function is used to alleviate the neuron death problem caused by the ReLU function and improve the robustness of the model. To further enhance the attention to citrus disease information and the ability to extract feature information, a multi-channel fusion backbone enhancement method (MCF) was designed in this work to process Dense Block. We use the 10-fold cross-validation method to conduct experiments. The average classification accuracy of DS-MENet on the dataset after adding noise can reach 95.02%. This shows that the method has good performance and has certain feasibility for the classification of citrus diseases in real life.

## Introduction

Citrus is one of the important economic crops in China It is rich in vitamin C, which is good for human health (Pujari et al., 2013). In recent years, affected by various environmental factors, citrus diseases have occurred frequently, causing huge economic losses. Therefore, the control of citrus diseases plays a very important role in citrus production. At present, the identification methods for citrus diseases in China are as follows (Zhang et al., 2018): (1) vegetable farmers roughly judge the disease type based on planting experience. (2) Professional technicians identify citrus pathogens. (3) They inhibit the development of citrus diseases by applying pesticides. The common problem of the first two methods is that it consumes a lot of manpower and time, and the efficiency is low. The third method will leave pesticides on the surface of citrus, causing environmental pollution. It can be seen that the traditional citrus disease detection methods cannot better ensure the quality of citrus and promote the economic development of agriculture. Therefore, it is necessary to develop a new citrus disease detection method to quickly and accurately identify and classify citrus diseases at an early stage and take effective disease control measures, thereby ensuring the quality and yield of fruits and reducing the economic losses caused by diseases to agricultural development.

As mentioned earlier, methods for artificially identifying citrus diseases generally suffer from inefficiencies and low accuracy. In recent years, the emergence and development of computer vision have brought new research directions for citrus disease detection. Machine learning is a commonly used technical means in computer vision, which is to improve the system’s performance by means of calculation and using existing experience. Support vector machine (SVM), cascaded Adaboost, and artificial neural network (ANN) are more commonly used methods in machine learning, of which the use of the cascaded Adaboost method can improve the recognition accuracy to more than 94% (Zhang et al., 2018). However, this method is only suitable for citrus disease images with simple backgrounds, and there are certain limitations in applying this method to complex backgrounds, such as actual farmland. In 2012, the research group of Hinton (Krizhevsky et al., 2012) proposed to use the deep convolutional neural network AlexNet for image recognition on the ImageNet dataset, which greatly reduced the classification error rate and set off a wave of deep learning. In recent years, deep learning has shown certain advantages in image recognition and classification, object detection, and other fields (Lin et al., 2021). In the study of citrus disease identification, deep learning can achieve high recognition accuracy (Pan et al., 2019); this is because the convolutional neural network (CNN) feature extraction layer can automatically learn features from citrus samples and extract useful feature information, but the acquisition of citrus disease images under different environmental conditions and the use of different network models for recognition will bring different classification results. Therefore, the main problems in the research are: (1) the process of image shooting will be interfered with by environmental factors such as light and background, resulting in poor original image quality of citrus, blurring, uneven brightness, and low color contrast, making it difficult to distinguish citrus disease characteristics. If the image is directly sent to the network for training without image preprocessing, the ability of the network to extract citrus disease features will be greatly weakened, resulting in low recognition accuracy. (2) There is a high similarity between some citrus diseases, such as citrus canker and citrus anthracnose. Traditional convolutional neural networks have difficulty distinguishing accurately, and the network model needs to be improved to improve the network’s ability to extract feature information.

To solve the problem wherein the disease characteristics are not obvious due to the interference of environmental factors such as external light in the process of shooting citrus images, some literature proposes to use Laplace filtering to enhance the edge information of the image and improve the clarity of the image. Experiments show that this method can better improve the phenomenon of image blur (Yao et al., 2017). The MSRCR algorithm (Rahman et al., 1996) is suitable for color image enhancement. It can enhance the details of dark parts by compressing the dynamic range of the image, but it is prone to color distortion. Dong et al. (2018) used homomorphic filtering to process the image, corrected the problem of uneven illumination of the image, and improved the visual effect of the image with low illumination, but the method was also prone to the phenomenon of over-enhancement, resulting in the image brightness being too high and some details being lost. Therefore, based on the ideas and advantages of the three algorithms of MSRCR, homomorphic filtering, and Laplacian filtering, this paper proposes an image enhancement algorithm for citrus diseases by HFLF-MS. First, we apply Laplacian filtering to the high-frequency components generated by the Homomorphic filtering; it is named Homomorphic Filtering Algorithm Optimized by Laplacian Filter (HFLF). Secondly, the images are processed separately using the MSRCR algorithm and the above-mentioned HFLF algorithm. Finally, the two images processed by the two algorithms are weighted and fused. The HFLF-MS algorithm can effectively improve the clarity and contrast of the image, enhance the details of the disease in the dark part, alleviate the problem of uneven brightness of the image, and provide a basis for the subsequent parts. To solve the problems of traditional convolutional neural networks in image recognition and classification, Zhang et al. (2018) proposed a deep learning model for citrus canker disease classification, which was improved based on lightweight AlexNet to achieve feature amplification and target optimization. Experimental results show that the model can efficiently and accurately classify citrus canker disease under the training condition of a small dataset. However, as the number of network layers deepens, the problem of gradient disappearance or gradient explosion will become more and more obvious, and in severe cases, the network will be stagnant, and the weights cannot be updated. In addition, when some citrus diseases have high similarities, higher requirements are also placed on the ability of the network to extract feature information. In this paper, a DS-MENet model was constructed to take DenseNet-121 as the primary network structure and replace traditional convolution in the Dense Block with depthwise separable convolution to reduce network parameters and reduce network running time. To alleviate the problem of neuron death caused by the ReLU activation function and improve the robustness of the model, an improved activation function ReMish was proposed. A multi-channel fusion backbone enhancement method (MCF) was proposed to enhance the backbone of Dense Block. This method solved the problem of low recognition accuracy caused by similar citrus disease characteristics, enhanced the attention to citrus disease information and feature information extraction ability, and improved the accuracy of citrus disease identification and classification. Therefore, this paper proposes a citrus disease identification method based on the combination of HFLF-MS and DS-MENet, and its main contributions are as follows.

(1) An HFLF-MS image enhancement algorithm is proposed. HFLF-MS first uses the MSRCR algorithm and the Homomorphic Filtering Algorithm Optimized by Laplacian (HFLF) algorithm to process the citrus disease images, respectively, and then weights the two images to form a new image. The MSRCR effectively enhances the details and contrast of the dark parts of the image, and the HFLF algorithm improves the clarity of the image and alleviates the problem of uneven illumination. The HFMS image enhancement algorithm can effectively enhance the dark detail features, color contrast, and sharpness of the image.
(2) The DS-MENet network model is proposed, which replaces the traditional convolution in DenseBlock with the depthwise separable convolution, which reduces the network parameters and reduces the running time of the network. A new activation function ReMish is proposed, which alleviates the neuron death problem caused by the ReLU function and improves the robustness of the model. To enhance the network’s ability to extract citrus disease features and improve the recognition and classification accuracy of similar disease features, a multi-channel fusion backbone enhancement method (MCF) was proposed to enhance the last convolutional layer of Dense Block. The core idea of this method is to group the feature images according to the number of channels and carry out convolution operations, respectively. In the process of operation, the feature images obtained after the convolution of two adjacent groups are fused as the convolution input of the next group to reduce the loss of the main feature information of the image.
(3) Compared with the traditional deep neural network model, higher recognition and classification accuracy can be obtained by using this method.

## Related work

The identification and classification of citrus diseases can be divided into two parts: image enhancement and image recognition.

Image enhancement is a key technology in recognition and classification work. The enhanced image effectively removes or weakens useless information, making it easier for machines to identify, and has been widely used in military and civilian fields. Li and Liu (2015) improved the image enhancement algorithm of the wavelet transform. The algorithm uses wavelet transform to separate the image and performs bilateral filtering and blurring transformation on low-frequency components and high-frequency components, respectively, which effectively enhances the image details. To improve the quality of low-illumination images, Zhang et al. (2016) proposed an image-enhancement algorithm based on K-means clustering. The improved algorithm divided the image into blocks and carried out histogram equalization of the information of each piece of image, thus improving the contrast and clarity of the image. Wei et al. (2019) proposed an image-enhancement algorithm based on the idea of fusion under Retinex theory, which performed MSR enhancement and single-scale Retinex enhancement based on bilateral filtering in YCbCr and RGB color space, respectively. Experimental results show that this algorithm can obtain images with better edge information and better detail information retention.

In the field of citrus disease image recognition, traditional machine learning methods and deep learning-based automatic feature extraction techniques have generally been used to classify citrus diseases. For traditional machine learning methods, handcrafted feature-extraction techniques with low computational complexity are used in most of the literature and have shown better performance (Janarthan et al., 2020). For example, Pydipati et al. (2015) proposed a machine learning method based on color symbiosis (CMM), created 4 feature models containing 39 variable texture feature sets, and classified these features using 4 methods. The classification accuracy of citrus diseases by using a neural network based on a back propagation algorithm and a neural network based on a radial basis function was more than 90%. However, this experiment was carried out in the laboratory with a controllable background environment, and the algorithm may greatly reduce the recognition accuracy if applied in the natural citrus forest. Wetterich et al. (2016) combined fluorescence imaging spectroscopy (FIS) and machine learning to accurately identify citrus diseases with similar symptoms. Mishra et al. (2012) used machine learning classification methods such as support vector machines to classify citrus canopy reflectance spectral data collected through near-infrared (VIS-NIR) spectroscopy. Deng et al. (2016) studied a classification of citrus Huanglongbing based on visible spectrum image processing and C-SVC (cost-support vector classification), using PCA to reduce the extracted features, and using the SVM classifier for classification. Good results are achieved at low cost and low computational complexity. However, due to the high variability of the canopy reflectance spectrum, multiple measurements of a single tree are required to obtain high classification accuracy. The main disadvantage of the manual feature extraction method is that the recognition accuracy is heavily dependent on the manually extracted feature parameters, which leads to the inability to achieve a high classification accuracy. In contrast, the automatic feature extraction technology based on deep learning has shown higher advantages. Zhang et al. (2018) proposed a deep learning model for citrus canker classification, which successfully achieved feature enhancement and target optimization, and used a small dataset for training and achieved high classification accuracy. Pan et al. (2019) proposed a mobile citrus disease diagnosis system, constructed six citrus disease image datasets, and used a simplified densely connected convolutional network (DenseNet) to identify and classify citrus diseases. Zhang et al. (2021) proposed an improved convolutional neural network combined with a state transfer algorithm (STA) to identify the surface defects of citrus, and the recognition accuracy of the trained model on the dataset can reach relatively high accuracy. Elaraby et al. (2022) used transfer learning to classify citrus diseases based on AlexNet and VGG19, and the proposed method used a momentum stochastic gradient descent algorithm (SGDM) for convergence speed. Janarthan et al. (2020) proposed a patch-based framework for citrus disease classification, consisting of an embedding module, a clustering prototype module, and a simple neural network classifier, which achieved promising results in terms of accuracy, parameter size, and time efficiency. Rehman et al. (2022) proposed a citrus disease classification method based on transfer learning and feature fusion, using Whale Optimization Algorithm (WOA) to obtain the best feature vector trained by MobileNetv2 and DenseNet201 for classification, and achieved high accuracy and computational efficiency. Sharif et al. (2018) proposed an automatic classification method for citrus diseases based on optimized weighted segmentation and feature selection, in which the optimized weighted segmentation algorithm extracted citrus lesions very efficiently, consisting of PCA scores, entropy and precision covariance vectors The hybrid feature selection method of can obtain the best features for post-classification. Syed-Ab-Rahman et al. (2021) proposed a two-stage deep CNN model. The two stages are to extract the potential disease target area and use the classifier to classify the potential target area. Effect. Xing et al. (2019) proposed a lightweight citrus pest and disease classification model based on Weakly DenseNet, using feature reuse and data augmentation to reduce the similarity between images, the algorithm improves parameter efficiency, and the model’s lightweight features can also be used for mobile application. In order to express the related research work more intuitively, we have listed a table including research methods, advantages and disadvantages. The specific details are shown in Table 1.

**TABLE 1:** Related research work details.

References	Method	Advantage	Drawback
Pydipati et al. (2015)	A machine learning method based on color symbiosis (CMM)	The classification accuracy obtained under controlled lighting conditions in an indoor laboratory is excellent	The model is sensitive to the background environment of citrus, and the classification of citrus diseases in natural environment may have limitations
Wetterich et al. (2016)	Identification method based on FIS (Fluorescence Imaging Spectroscopy) and machine learning	High accuracy for classification of similar diseases of citrus (canker and scab)	High requirements for image quality
Mishra et al. (2012)	Classification of Citrus Diseases Based on Support Vector Machines and VIS-NIR (Near-Infrared Spectroscopy)	Classification algorithms are common and effective	The experimental data has strong uncertainty and is greatly affected by the environment
Deng et al. (2016)	Identification method of citrus HLB disease based on visible spectrum image processing and C-SVC (cost-support vector classification)	(a) Low computational complexity and high efficiency (b) Low cost	Classification accuracy needs to be improved
Zhang et al. (2021)	Optimized AlexNet citrus disease classification model	The network structure is simple, the number of parameters is small, and the performance is relatively excellent	There are certain challenges in applying to multi-classification of citrus diseases
Pan et al. (2019)	Intelligent Diagnosis System of Citrus Diseases Based on Mobile Service Computing	(a) WeChat applet is rich in functions, convenient and practical (b) Simplified DenseNet can reduce prediction time	Disease identification accuracy needs to be improved
Elaraby et al. (2022)	A citrus disease classification model based on transfer learning and optimized SGDM (Stochastic Gradient Descent with Momentum)	(a) Stochastic Gradient Descent with Momentum (SGDM) can speed up convergence? (b) Transfer learning can improve training efficiency	The classification of similar diseases will have certain limitations
Janarthan et al. (2020)	A Patch-Based Framework for Citrus Classification	(a) Fast and accurate classification can be achieved with sparse data (b) Model computational complexity is low and lightweight	The classification accuracy of similar diseases needs to be improved
Rehman et al. (2022)	A classification method of citrus diseases based on transfer learning and feature fusion	(a) Image preprocessing with hybrid contrast stretching can improve image quality (b) The feature set obtained by fusing different networks can deeply extract image information (c) Whale Optimization Algorithm (WOA) is used to select salient features, which can reduce computation time	Lack of performance comparisons with more state-of-the-art classification models
Sharif et al. (2018)	Automatic classification of citrus diseases based on optimized weighted segmentation and feature selection	The optimized weighted segmentation algorithm can segment the lesions, which is beneficial to the extraction of later feature information	Less suitable for citrus diseases without lesions, such as citrus Huanglongbing
Syed-Ab-Rahman et al. (2021)	A classification model of citrus diseases based on two-stage deep CNN	Feature sharing can be achieved between the two stages to reduce model training overhead	The robustness of the model needs to be further improved
Xing et al. (2019)	A lightweight citrus pest identification model based on Weakly DenseNet	(a) Feature reuse and data augmentation algorithms can reduce similarity between images (b) Lightweight design for easy use in mobile apps	The proportion of the object to be classified in the image will affect the output result

**FIGURE 1:**
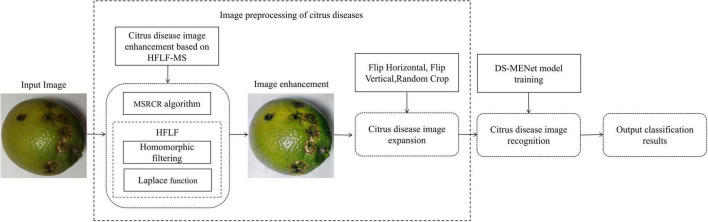
Working principle diagram of the system.

Although deep learning has achieved success in the field of citrus disease identification in the above studies, there is still room for improvement in the identification accuracy, and the training time of the model needs to be reduced as much as possible. The existing citrus disease classification methods are mainly based on samples with better image quality. However, if citrus is photographed in real life, it is easily affected by environmental factors such as light, the images generally have the phenomenon of inconspicuous dark details, low color contrast, and blurring. In addition, there is a high similarity between samples of some categories of citrus diseases, such as canker, scab, and anthracnose. These factors will increase the difficulty of later identification and classification. To achieve a more accurate classification of various diseases, it is necessary to retain the original detailed information as much as possible and strengthen the ability to extract image feature information. Therefore, this paper proposes a citrus disease identification method that combines HFLF-MS and DS-MENet, and the working principle is shown in Figure 1. To improve the image quality and make the citrus disease features more obvious, the HFLF-MS algorithm is used to process the images. After preprocessing, the images are sent to DS-MENet for training and testing. In DS-MENet, depthwise separable convolution is used to replace the traditional convolution in Dense Block to reduce network parameters and model running time; the new activation function ReMish is used to alleviate the neuron death problem caused by the original ReLU function and enhance the robustness of the model: the MCF method is used to enhance the backbone of the Dense Block, improve the network’s ability to extract citrus disease features, and preserve detailed information to the greatest extent. Experimental results show that, compared with modern convolutional neural network models, such as AlexNet (Krizhevsky et al., 2012), ResNet50 (He et al., 2016), ResNeXt (Gp et al., 2020), InceptionV4 (Tian et al., 2021), MobileNetV3 (Tarek et al., 2022), EfficientNet (Chen et al., 2021), DenseNet121 (Huang et al., 2017), and EfficientNetV2 (Sunil et al., 2022), the method presented in this paper has a better recognition and classification effect on citrus diseases with similar characteristics.

## Materials and methods

### Data acquisition

**FIGURE 2:**
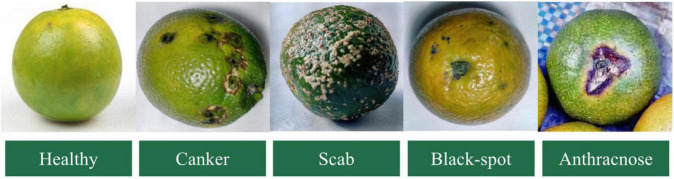
Collection of citrus disease images.

Data collection is an indispensable part of citrus disease identification and classification. The data set in this paper comes from field shooting and the Internet. The citrus images taken on the spot are from the National Citrus Virus-Free Original Seedling Cultivation Base in Changsha City, Hunan Province, and were taken with a Canon EOS R6 camera with a resolution of 5,472 × 3,648 under natural light. The images obtained through the Internet are derived from the open-source datasets of the kaggle platform: PlantifyDr Dataset, Dataset for Classification of Citrus Diseases, Fruit Diseases. The training of the network requires a large number of data sets, so after enhancing the collected images, the number of data sets is expanded by horizontal flipping, vertical flipping, and random cropping, and finally, 9,258 images are obtained, all in jpg format to save. Table 2 shows the number and proportion of various citrus disease images and healthy citrus images. Disease types include citrus canker, scab, black spot, and anthracnose, a total of four diseases, as shown in Figure 2.

**TABLE 2:** Number and proportion of citrus diseases.

Disease type	Original number	Expanded number	Percentage (%)
Healthy	564	1,692	18.3
Canker	634	1,902	20.5
Scab	592	1,776	19.2
Black spot	743	2,229	24.1
Anthracnose	553	1,659	17.9

Next, we will elaborate on the image enhancement algorithm and citrus disease classification model proposed in this paper. For ease of reading, we list the variables included in the formulas that appear below, as shown in Table 3.

**TABLE 3:** Variable names and their meanings.

Variable	Meaning
*i*	color channel, *i* ∈ *R*, *G*, *B*
*i*(*x*, *y*)	luminance component
*r*(*x*, *y*)	reflection component
*f*(*x*, *y*)	input image
*r*_*i*_(*x*, *y*)	Output of the ith channel SSR
*F*(*x*, *y*)	Gaussian wrap function
*c*	Gaussian Surround Scale
*r_MSR_i__*(*x*, *y*)	the result of MSRCR enhancement of the ith color channel
*F*_*n*_(*x*, *y*)	Gaussian wrapping function at the nth scale
ω_*n*_	Weight coefficient for the nth scale
*N*	total number of scales, N=3
*r_MSRCR_i__*(*x*, *y*)	the result of MSRCR enhancement of the ith color channel
*C*_*i*_(*x*, *y*)	color restoration factor of the ith color channel
α	controlled nonlinear intensity
β	Gain constant
*Y*	Parameter quantity
*H*	Length of the convolution kernel
*W*	Width of the convolution kernel
*C*	channel number of the input feature map
*K*	channel number of the output feature map
*Y_DW_*	The parameter quantities of DW convolution
*Y_PW_*	The parameter quantities of PW convolution
*b*_*i*_	Input feature maps of the ith group
*a*_*i*_	The ith group of feature maps
*f_sq_*	The average pooling function
*f_ex_*	The excitation function
*f_scale_*	The multiplication function

### Image enhancement of citrus diseases based on weighted fusion algorithm combining MSRCR and homomorphic filtering optimized by Laplacian filter

During the shooting of citrus disease images, the image quality is easily disturbed by various environmental factors such as light, resulting in poor image quality, uneven illumination, indistinct disease features in dark parts, blurring, and low color contrast. These problems will adversely affect the training and testing of the later network and increase the difficulty of identification and classification. To improve the reliability and accuracy of post-recognition classification, the image needs to be enhanced. Therefore, this paper uses the HFLF-MS algorithm to enhance the citrus disease images. The algorithm first uses the MSRCR algorithm and the HFLF algorithm to process the images, respectively, and then weights and fuses the two processed images to obtain the enhanced image. The MSRCR algorithm effectively enhances the dark details and color contrast of the image. The HFLF algorithm combines homomorphic filtering and Laplace filtering to improve the clarity of the image and alleviate the phenomenon of uneven illumination. The weighted fusion image method overcomes the shortcomings of using the MSRCR algorithm or homomorphic filtering alone for image enhancement and can better describe the details of the image.

**FIGURE 3:**
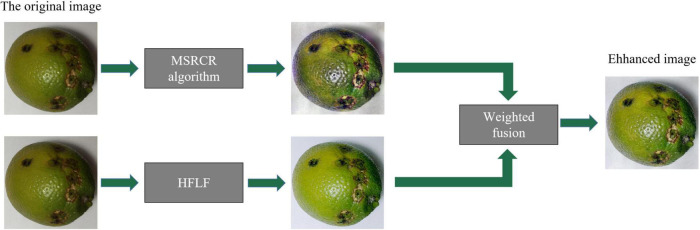
HFLF-MS working principle diagram.

The HFLF-MS algorithm can not only highlight the details of the image well, but the enhanced image also has natural color and a good visual effect. The specific workflow of the HFLF-MS image enhancement algorithm is shown in Figure 3.

#### Multi-scale retinex with color restoration

When the overall color contrast of the image is low and the dark details are not obvious or missing, the color recovery algorithm based on Retinex (Land Edwin, 1978) theory can effectively enhance the image to improve the color contrast and highlight the dark details of the image. The MSRCR algorithm is a color-restoration algorithm with good effect, which is derived from the color-restoration algorithm based on Retinex theory.

In the Retinex theory, an image can be regarded as the product of the luminance component and the reflection component, represented by i(*x*, *y*) and r(*x*, *y*), respectively. The mathematical model is Formula (1). In Formula (1), the luminance component i represents the incident light image, and the reflection component r represents the reflection property of the object, that is, the intrinsic property of the image. The purpose of the Retinex algorithm to enhance the image is to remove the brightness component i from image f, and obtain the reflection component rto the greatest extent.


(1) f(x,y)=i(x,y)⋅r(x,y)

The SSR algorithm is proposed based on the Retinex theory (Hines et al., 2005), and the algorithm can be expressed as Formulas (2)–(4). Among them,   r_*ij*_ (*x*, *y*) is the output image of SSR, F(*x*, *y*) is the Gaussian wrapping function, and *c* is the Gaussian wrapping scale.


(2) $$r_{i}\left(x,y\right)={\mathrm{logf}}_{i}\left(x,y\right)-\log\left[F\left(x,y\right)f_{i}\left(x,y\right)\right]$$

(3) $$F\left(x,y\right)=\text{μ}\exp\left[\frac{-{\left(x^{2}+y^{2}\right)}^{2}}{c^{2}}\right]$$

(4) ∫∫F(x,y)dxdy=1

It can be seen from Formulas (2) and (3) that the SSR algorithm estimates the illumination change in the image by calculating the pixel point and the surrounding area under the action of weighted average, removes i(*x*, *y*), and retains f(*x*, *y*).

The multi-scale Retinex algorithm (MSR) is developed based on SSR. It linearly weights multiple color channels with a fixed number of scales. The calculation formula of the algorithm is as follows. r_*MSR_i_*_ (x,y) is the result obtained by the MSR algorithm for the ith color channel, *i* ∈ *R*, *G*, *B*, represents the three color bands of the image, F_*n*_ (*x*, *y*) is the Gaussian function of the nth scale, _ω_*n*__ is the weight of the result at the nth scale, and satisfies the normalization condition: $\sum_{n=1}^{N}\omega_{n}=1$, N is the total number of scales, where N=3, represents a color image.


(5) $$r_{{\mathrm{MSR}}_{i}}\left(x,y\right)=\sum_{n=1}^{N}\omega_{n}\left\{{\mathrm{logf}}_{i}\left(x,y\right)-\log\left[F_{n}\left(x,y\right)f_{i}\left(x,y\right)\right]\right\}$$

Although MSR can simultaneously satisfy color fidelity and detail enhancement, in the process of image enhancement, the problem of local color distortion of the image will still occur due to the increase in noise, which reduces the overall visual effect. The Multiscale Retinex with Color Recovery (MSRCR) algorithm can improve this problem. Based on MSR (Rahman et al., 1996), this algorithm makes up for the defect of color distortion by introducing a color recovery factor and preserves the original image details to the greatest extent while maintaining the original color of the image. The expressions are Formulas (6) and (7). For Formulas (6) and (7), *r*_*MSRCR*_*i*__(*x*, *y*) is the result of MSRCR enhancement of the jth color channel, and *c*_*i*_(*x*, *y*) is the color restoration factor of the ith color channel, α is the controlled nonlinear intensity, β is the gain constant, and f_*j*_ (*x*, *y*) is the distribution of the citrus disease image in the jth color channel.


(6) $$r_{{\mathrm{MSRCR}}_{i}}\left(x,y\right)=C_{i}\left(x,y\right)\cdot r_{{\mathrm{MSR}}_{i}}\left(x,y\right)$$

(7) $$\begin{array}{c} C_{i}\left(x,y\right)=\beta \left\{\log\left[\text{α}f_{i}\left(x,y\right)\right]-\log\left[\sum_{i=1}^{N}f_{i}\left(x,y\right)\right]\right\} \end{array}$$

The MSRCR algorithm adjusts the proportional relationship between the three color channels in the original image through the color restoration factor, highlighting the details in the darker areas in the image, and the local color contrast is also improved.

#### Homomorphic filtering optimized by Laplacian filter

In the process of image acquisition, due to the influence of environmental factors such as light and weather, low-illumination problems such as backlight will inevitably occur, and the image is prone to blurring and uneven brightness, which makes it difficult to distinguish disease characteristics. The HFLF algorithm can effectively solve such problems. The HFLF algorithm combines homomorphic filtering and Laplacian filtering and performs a Laplacian filtering on the obtained high-frequency components in the process of homomorphic filtering. Homomorphic filtering is a commonly used low-illumination image enhancement method. Its core idea is to suppress the low-frequency luminance component to correct the uneven illumination and enhance the high-frequency reflection component to enhance the low-illumination details of the image. Laplacian filter is a commonly used two-dimensional linear filter, which uses the Laplacian operator to enhance the feature points of the image to achieve the effect of image sharpening.

The HFLF algorithm adopts the illuminance-reflection model of the image represented by the Formula (1). First, we can take the logarithm of both sides of Formula (1), convert the multiplication operation into an addition operation, and obtain Formula (8). Afterward, Fourier transform is performed on Formula (8) to obtain Expression (9) in the frequency domain. Among them, I(*u*, *v*) is the low-frequency part corresponding to the illumination component, and R(*u*, *v*) is the high-frequency part corresponding to the reflection component (Yu et al., 2021). The high-frequency part mainly reflects the edge information of the image.


(8) lnf(x,y)=lni(x,y)+lnr(x,y)

(9) F(u,v)=I(u,v)+R(u,v)

Secondly, to make the edges, contours, and details of citrus disease images clearer, the Laplacian operator filters the high-frequency parts (Khan et al., 2020). We can denote ∇^2^*r* (*x*, *y*) as the Laplacian operator, and (x,y) as the image plane coordinates of the high-frequency part of the pixel, and the Laplacian operator can be defined as:


(10) $$\nabla^{2}r\left(x,y\right)=\frac{\partial^{2}r}{\partial x^{2}}+\frac{\partial^{2}r}{\partial y^{2}}=r\left(x-1,y\right)+r\left(x,y+1\right)+r\left(x+1,y\right)+r\left(x,y-1\right)-4r\left(x,y\right)$$

**FIGURE 4:**
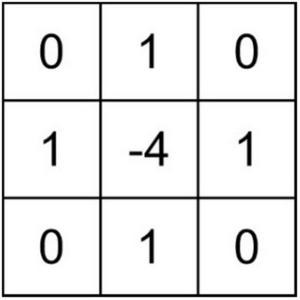
Laplacian filter template.

According to Formula (10), the value filtered by the Laplacian operator is the sum of four times the gray value of the center pixel and the gray value of the four upper, lower, left, and right pixels. The Laplacian filter template is a fixed 3 × 3 size window, as shown in Figure 4, that is, the four-neighborhood Laplacian filter template.

By traversing any pixel of the image corresponding to the high-frequency part of the four-neighborhood Laplacian filter template, the Laplacian filter value of each pixel can be obtained. It is important to note that the Laplace sharpening result of the high-frequency part is G(*u*, *v*); then, the expression is:


(11) $$\begin{array}{c} G\left(u,v\right)=R\left(u,v\right)-F\left[\nabla^{2}r\left(x,y\right)\right] \end{array}$$

After the high-frequency part is subjected to Laplace filtering, the homomorphic filter function H(*u*, *v*) filters the high- and low-frequency components. The traditional H(*u*, *v*) is often a Butterworth filter function, and its expression is Formula (12). r_*h*_ and r_*l*_ are the multiples of high-frequency component enhancement and low-frequency component suppression, respectively; c is the sharpening coefficient; D_0_ is the cutoff frequency radius; and D(*u*, *v*) is (*u*, *v*) to the distance of the filter center.


(12) $$\begin{array}{c} H\left(u,v\right)=\frac{r_{h}-r_{l}}{1+{\left[c\cdot \frac{D_{0}}{D\left(u,v\right)}\right]}^{2n}}+r_{l} \end{array}$$

The traditional Butterworth filter function deals with low frequency and high frequency in the same way. To better improve the phenomenon of uneven illumination and low illumination and enhance the details of the dark part of the image, two improved exponential homomorphic filter functions are selected in this paper, which can process the low-frequency and high-frequency components separately. It is important to note that the filtered result is H_*f*_ (u,v), and the improved homomorphic filtering function and filtering method are as follows:


(13) $$H_{h}\left(u,v\right)=\left(r_{h}-r_{l}\right)\cdot exp{\left(-c\cdot \frac{D_{0}}{D\left(u,v\right)}\right)}^{n}+r_{l}$$

(14) $$H_{l}\left(u,v\right)=1-\left[\left(r_{h}-r_{l}\right)\cdot \exp{\left(-c\cdot \frac{D_{0}}{D\left(u,v\right)}\right)}^{n}+r_{l}\right]$$

(15) $$H_{f}\left(u,v\right)=H_{l}\left(u,v\right)I\left(u,v\right)+H_{h}\left(u,v\right)G\left(u,v\right)$$

After the high- and low-frequency components are filtered by the homomorphic filter functions H_*h*_ and H_*l*_, the inverse Fourier transform is performed on both sides of Formula (15) at the same time, and Formula (16) can be obtained. Finally, we can take the exponent of Formula (16). We can denote z(x,y) as the image processed by the HFLF algorithm, and the expression is Formula (17).


(16) $$h_{f}\left(x,y\right)=h_{i}\left(x,y\right)+h_{g}\left(x,y\right)$$

(17) $$z\left(x,y\right)=e^{h_{f}\left(x,y\right)}=e^{h_{i}\left(x,y\right)}e^{h_{g}\left(x,y\right)}$$

#### Weighted fusion

The purpose of image fusion is to synthesize the results obtained by processing the same image with multiple algorithms so that the synthesized image can highlight the details of the image to the greatest extent. Weighted average fusion is a simple and commonly used image fusion method. Its basic idea is to perform a weighted average of the corresponding pixels of multiple images under the condition that the weighting coefficient is the best and then perform fusion.

The image processed by the MSRCR algorithm can be obtained from Formula (6), denoted as M(*i*, *j*), and the image processed by the HFLF algorithm can be obtained from Formula (17), denoted as N(*i*, *j*). K(*i*, *j*) represents the fused image, and i and j represent the coordinates of the pixels in the image. The weighted image fusion can be expressed as:


(18) $$\begin{array}{c} K\left(i,j\right)=\text{μ}M\left(i,j\right)+\left(1-\mu \right)N\left(i,j\right) \end{array}$$

Among them, β is the weighting coefficient, and 0 ≤ β ≤ 1, the size of β can be adjusted according to the actual needs. Through experiments on many pictures, it can be concluded that the range of the optimal weighting coefficient is 0.4–0.6. After the enhancement, the obtained image can take into account the advantages of the MSRCR algorithm and the HFLF algorithm simultaneously, overcome the shortcomings of the two algorithms, and have a better visual effect.

#### HFLF-MS algorithm summary

Based on the principles of MSRCR, HFLF and weighted fusion proposed above, the flow of the HFLF-MS algorithm can be summarized as follows:

Step 1: the input color image is processed by the MSRCR algorithm. Decompose the input color image I(x,  y) into three RGB images, and convert its data type to double type. The following processes each image separately (using R as an example):

(1) Determine the Gaussian surround function according to formulas (2) and (3), calculate the corresponding Gaussian template, and select the value of scale c to be 250.
(2) According to formula (5), the weighted average of the results obtained under the three scales is calculated, and the value of the weight coefficient w is 0.33, 0.33, and 0.34, respectively.
(3) The color restoration factor is calculated according to formula (7), the contrast is stretched and enhanced on the image, and then   r_*MSRCR_i_*_ (*x*, *y*) can be calculated by substituting formula (6).

(1)–(3) are performed on the three channels of R, G, and B, respectively, and finally integrated into a complete image I_*MSRCR*_ (x,y).

Step 2: Decompose the input color image I (x, y) into three RGB images, and convert the data type to double. The following processes each image separately (using R as an example):

(1) Enhance the high-frequency area of the image, that is, perform Laplace sharpening enhancement.
(2) Determining the image in the frequency domain by taking the logarithm and Fourier transform of the input.
(3) Determine the Basworth function according to formulas (13) and (14), and set r_*h*_ and   r_*l*_ to be 1.1 and 0.01, respectively.
(4) Multiplying the Butterworth function with the frequency domain output.
(5) Inverse Fourier transform of the filtered frequency domain image to time domain.
(6) Exponentiate an image in the time domain

(1)–(6) are performed on the three channels of R, G, and B, respectively, and finally integrated into a complete image I_*HFLF*_ (x,y).

The third step: weighted fusion of I_*MSRCR*_ (x,y) and I_*HFLF*_ (x,y) according to formula (18), take μ = 0.3. The final image enhanced by HFLF-MS algorithm is obtained.

**FIGURE 5:**
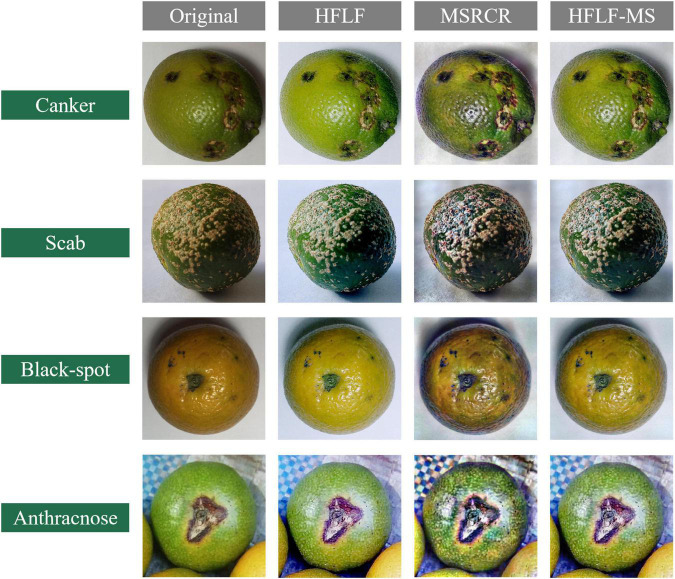
Enhancement of citrus disease images.

As shown in Figure 5, the four images are the original image, the image processed by MSRCR, the image processed by HFLF, and the image enhanced by HFLF-MS.

### Identification of citrus disease based on DS-MENet model

Since some citrus diseases will show similar characteristics, it is necessary to strengthen the feature extraction ability of the convolutional neural network to obtain more detailed features and reduce the false-positive rate. When the traditional convolutional neural network is used for feature extraction, with the deepening of the network, it is easy to cause the loss of important features, the disappearance of the gradient, or the explosion of the gradient, which affects the classification effect. Therefore, this paper selects DenseNet-121 as the main body of the network to construct our neural network framework. Dense Block is the core component of the DenseNet-121 network, which can effectively obtain the main features of the image. The basic Dense Block structure is relatively simple, and the feature information extraction ability needs to be further enhanced.

To improve the overall learning effect of the network, this paper replaces the traditional convolution of Dense Block with depthwise separable convolution to reduce network parameters. To improve the robustness of the model, a new activation function ReMish is proposed to alleviate the problem of neuron death caused by the original ReLU function. In addition, we propose an MCF method based on grouped convolution and multi-channel fusion, which is used to enhance the backbone of the Dense Block to improve the network’s ability to extract citrus disease features. DS-MENet has good robustness, which can effectively improve the recognition and classification accuracy of similar disease features and reduce the running time of the model.

**FIGURE 6:**
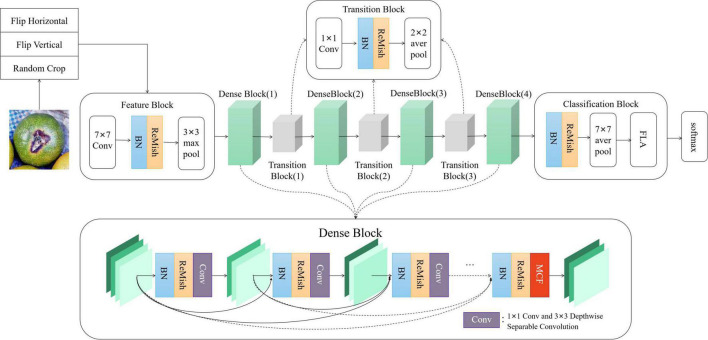
DS-MENet network structure.

**FIGURE 7:**
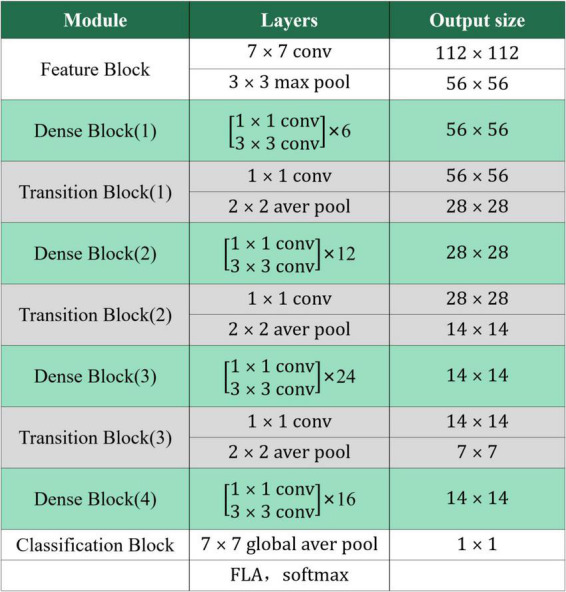
Output parameters of each layer of DS-MENet.

The DS-MENet network structure contains one Feature Block, one Classification Block, three Transition Blocks, and four Dense Blocks. The network structure of DS-MENet and the output results of each layer are shown in Figures 6, 7, respectively. The main workflow is as follows:

(1) The first part of DS-MENet is Feature Block, which contains 7 × 7 convolutional layers and 3 × 3 max pooling layers. The BN layer and the ReMish activation function are added between the convolutional layer and the pooling layer. The BN layer can enhance the generalization ability of the model and speed up the convergence speed of the network. The application of the ReMish activation function can improve the robustness of the network.
(2) The second part of DS-MENet is Dense Block (1), which consists of 6 layers, including 5 convolution layers composed of 1 × 1 convolution, 3 × 3 depth separable convolution, and 1 convolution layer based on MCF backbone enhancement. BN layer and ReMish activation function are also included between each convolution layer. As shown in Figure 6, Dense Block directly connects all the layers, and each layer uses the features of all previous layers as input before feature transfer, and transmits its own output feature map to all subsequent layers. This structure reduces network parameters, strengthens the utilization and transfer of features, and solves the problem of gradient disappearance or gradient explosion caused by network deepening. The use of depthwise separable convolution reduces network parameters. The MCF method is applied to the last set of convolutional layers, which can further enhance the main feature information of the feature map.
(3) The third part is Transition Block, which contains a 1 × 1 convolutional layer, BN layer, ReMish, and 2 × 2 average pooling layer. Transition Block is used to compress the number of output channels of Dense Block and reduce network parameters.
(4) The part after DS-MENet is to cycle through three Dense Blocks and two Transition Blocks. The structure of the Transition Block is the same as the third part above. The Dense Block (2), Dense Block (3), and Dense Block (4) have 12, 24, and 16 layers, respectively. The last layer is the convolution layer based on MCF backbone enhancement, and the other layers are the convolution layer composed of 1 × 1 convolution and 3 × 3 depth separable convolution.
(5) The last Dense Block is followed by the Classification Block, which consists of BN, ReMish, 7 × 7 average pooling layers, and linear layers.
(6) The last part of DS-MENet is to determine the classification results of the input citrus disease images through the softmax activation function.

#### DenseNet

As the number of convolutional neural network layers increases, the problem of vanishing gradients is easy to occur. In order to solve this problem, ResNet (He et al., 2016) connects the bypass information and shortens the network by randomly dropping some layers in the process of network training, so as to achieve a better training effect. DenseNet was proposed in 2017 (Huang et al., 2017). Compared with the classic ResNet and GoogleNet, DenseNet is separated from the stereotyped thinking of deepening the number of network layers and widening the network structure. From the perspective of features, a new dense connection mechanism is proposed. The layers are cascaded, each layer of features accepts the input from the previous layers, and transmits its own feature map to all subsequent layers, which makes the transfer of feature information more effective. DenseNet can optimize the transfer of information and gradients in the network. Each layer can directly use the gradient of the loss function and the initial input information for deep supervision, which is very helpful for training deep networks. In addition, the dense connections of the DenseNet network also have a regularization effect, which is beneficial to reduce the problem of overfitting during training. Since we are not studying a very complex multi-classification problem, considering the computational cost, this paper selects DenseNet-121 as the backbone network for citrus disease classification.

#### Depthwise separable convolution

To further reduce the network parameters in the process of citrus disease identification, this paper replaces the 3 × 3 ordinary convolution in each Dense Block with a depthwise separable convolution. Depthwise separable convolution is the core idea of MobileNet (Howard et al., 2017), This is a method that separates the regions and channels of the feature map and decomposes the standard convolution into DW convolution and PW convolution in a factorized manner.

**FIGURE 8:**
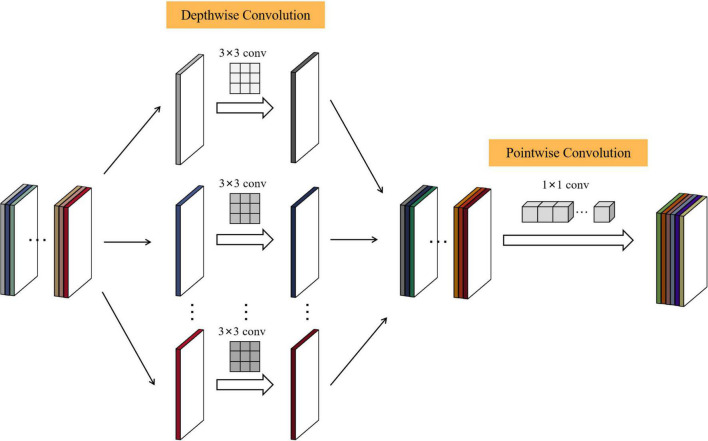
Working principle of depthwise separable convolution.

Taking the first Dense Block as an example, according to the output results of each layer of DS-MENet shown in Figure 7, the dimension of the citrus disease feature map input to the first 3 × 3 convolutional layer is 56 × 56 × 128. In ordinary convolution, the feature map is directly convolved with 32 3 × 3 convolution kernels, padding and stride are set to 1, and the output dimension is 56 × 56 × 32. In deeply separable convolution, DW convolution is performed first, and a convolution kernel is responsible for one channel of the feature graph. The feature graph of 56 × 56 × 128 was convolved with 128 3 × 3 convolution kernels. The padding and stride were set to 1, and finally obtained a feature graph with output of 56 × 56 × 128. Then, we performed PW convolution on the feature map, that is, perform convolution operation with 32 1 × 1 convolution kernels, with padding and stride also set to 1, and finally obtain a feature map with an output dimension of 56 × 56 × 32. The specific process of depthwise separable convolution is shown in Figure 8.

Compared with ordinary convolution, the biggest advantage of depthwise separable convolution is that it can reduce the network parameters exponentially, thereby reducing the network running time. The calculation formula for calculating the number of ordinary convolution parameters is as follows:


(19) $$\begin{array}{c} Y=H\times W\times C\times K \end{array}$$

Among them, Y is the parameter quantity, H is the length of the convolution kernel, W is the width of the convolution kernel, C is the channel number of the input feature map, and K is the channel number of the output feature map. In an ordinary 3 × 3 convolution operation, the corresponding regions of the feature map and all channels need to be considered simultaneously. When the dimension of the input image is 56 × 56 × 128, the parameter amount can be obtained as 36,864 according to Formula (19).

Since the depthwise separable convolution divides the convolution into two processes: DW convolution and PW convolution, the calculation formula of the parameter quantity is somewhat different from that of ordinary convolution. The specific calculation method is as follows:


(20) $$Y_{\mathrm{DW}}=H\times W\times C$$

(21) $$Y_{\mathrm{PW}}=H\times W\times C\times K$$

Among them, Y_*DW*_ and Y_*PW*_ are the parameter quantities of DW convolution and PW convolution, respectively, and the definitions of other parameters are the same as Formula (19). Under the same input conditions, the parameter amount of the DW convolution stage is 1,152, and the parameter amount of the PW convolution stage is 4,096; that is, the overall parameter amount is 5,248, which is about 1/7 of the ordinary convolution parameter amount. It can be seen that compared with ordinary convolution, depthwise separable convolution can greatly reduce the number of parameters of the network, which can reduce the running time.

#### Multi-channel fusion backbone enhancement

In the classification task of citrus disease images, it is inevitable that the image categories are different but the features are similar, which puts forward higher requirements for the ability of the network to extract feature information. Feature fusion is beneficial to retain a large amount of image information, thereby improving the accuracy of image classification. At present, there are many implementation methods for feature fusion, which fuse feature information from different perspectives and ideas, and achieve good results. The design of the DenseNet network structure is actually a simple feature fusion, which encourages feature reuse, and enables the shallow feature information to be directly used by the deep layer through the cascade. However, DenseNet also has shortcomings. Each layer of DenseNet simply combines the feature maps obtained from the previous layers, so the ability to extract feature information still needs to be further improved. In response to this problem, some scholars have proposed other new methods of feature fusion based on DenseNet. Zhai et al. (2020) proposed a new feature fusion module based on the traditional FPN pyramid structure and applied it to DenseNet. This method fuses shallow feature maps with high resolution but weak semantic information and Deep feature maps with high semantic information but low resolution, so the context information is more effectively utilized. Pearline and Vajravelu (2019) and Montalbo (2021) proposed that DenseNet and other network models extract features from images separately, and then connect different feature vectors.

In the above research, although the mentioned methods basically fuse multiple feature maps directly, it is not considered that with the increase of the depth of the DenseNet network, the channel information of the output feature map is not fully utilized. Therefore, in order to strengthen the utilization of feature map channel information, this paper proposes the MCF algorithm to enhance the backbone of the Dense Block, which significantly improves the ability to utilize the channel feature information and solves the problem that the network does not fully utilize the convolution kernel channel information in the process of gradually deepening.

**FIGURE 9:**
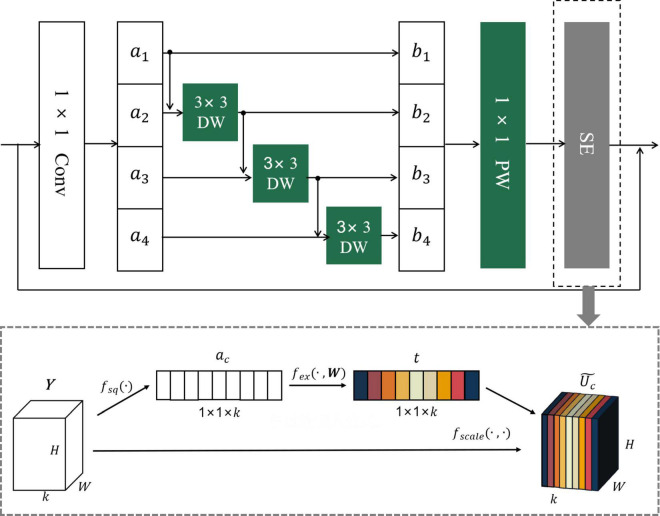
Working principle diagram of MCF algorithm.

The MCF backbone enhancement method is mainly divided into two parts, namely, grouped convolution based on multi-channel fusion and attention mechanism. In the process of network training and testing, ordinary convolution will generate a high amount of parameters and cannot make full use of the channel information of the convolution kernel. Therefore, the original ordinary convolution is replaced by a depthwise separable convolution, and the method of grouping convolution and feature map fusion between adjacent channels is adopted to enhance the utilization of feature information. This method cannot only reduce the number of parameters generated in the convolution process but also the inter-correlation between channels can fully extract the detailed information of citrus disease features. Attention mechanism is an important concept in the field of deep learning and has gradually become an important part of the neural network structure. SE is a typical attention mechanism, which can enhance the degree of attention to the local information of citrus disease images, explicitly establish the interconnection between the feature map channels, and ensure the lossless transmission of the original information (Hu et al., 2017). Figure 9 shows the overall architecture of the MCF.

The specific implementation steps of the MCF backbone enhancement method are as follows:

(1) First, record the input of the last set of convolutional layers as X_0_ (H × W × C); X_0_ is convolved with m 1 × 1 × C convolution kernels, and the feature map X′ with dimension H × W × m is generated.
(2) Secondly, X′ is equally divided into 4 groups according to the number of channels, marked as a_1_, a_2_, a_3_, a_4_, and the size of each group of feature maps is H × W × $\frac{m}{4}$.
(3) After that, DW convolution is performed on each group separately, and one convolution kernel corresponds to one channel of the feature map. As can be seen from Figure 9, the input parts of the DW convolution of different groups are different, except that the input of the DW convolution of the first group is a_1_ *a*_1 obtained after grouping, and the input of each subsequent group of DW convolution is a_*i*_ + *b*_*i*−1_(1 < *i* < 4), which means that the input of each group depends on the output obtained by the DW convolution of the previous group, and a_1_*a*_1 can be expressed by Formula (16). Due to the design of this grouping structure, the number of 3 × 3 convolution kernels in each group of DW convolutions varies. According to the basic principle of the above-mentioned depthwise separable convolution, it can be obtained that the number of 3 × 3 convolution kernels in each group is $\frac{m}{4}$ i, and the output b_*i*_ dimension after DW convolution is H × W × $\frac{m}{4}$ i.

(22) $$\begin{array}{c} b_{i}=\left\{ \begin{array}{c} a_{i},i=1\\ Conv_{\mathrm{DW}3\times 3}\left(a_{i}+b_{i-1}\right),1<i<4 \end{array}\right. \end{array}$$

(4) After DW convolution, PW convolution is performed on *b*_*i*_; that is, each group is convolved with $\frac{k}{4}$ convolution kernels of 1 × 1 × $\frac{m}{4}$ i to obtain the output of each group $b{}_{i}^{\prime }$; The dimensions are H × W × $\frac{k}{4}$, and k is the number of channels of the target output feature map. The output $b{}_{i}^{\prime }$ of each group is superimposed to obtain the feature map Y (H × W × k). Steps (3) and (4) replace the traditional 3 × 3 convolution with depthwise separable convolution. This improved method can effectively expand the network width, and the feature information of the original image has been effectively enhanced and utilized.
(5) Finally, Y is fed into the SE module to obtain the final output $\tilde{U_{c}}$. The working steps of the SE attention mechanism are as follows:

a. First, the global information of each feature map channel is extracted using global average pooling, and Y is compressed by the spatial dimension H × W to generate a feature vector a_*c*_ (1 × 1 × k). The calculation formula of a_*c*_ is as follows:

(23) $$\begin{array}{c} a_{c}=f_{\mathrm{sq}}\left(Y\right)=\frac{1}{H\times W}\sum_{i=1}^{H}\sum_{j=1}^{W}Y\left(i,j\right) \end{array}$$

where *f*_*sq*_ is the average pooling function and c is the channel index.

b. Secondly, a_*c*_ passes through the fully connected layer, the ReMish activation function, the fully connected layer, and the sigmoid activation function in turn. In this process, the correlation between channels is completely captured, ensuring that multiple channels are emphasized, and finally, the channel weight vector t (1 × 1 × k). The expression for *t* is:

(24) $$\begin{array}{c} t=f_{\mathrm{ex}}\left(a,W\right)=\sigma \left(W_{2}\text{δ}\left(W_{1}a\right)\right) \end{array}$$

Among them, δ is the ReMish activation function, σ is the sigmoid function, f_*ex*_ is the excitation function, W_1_ and W_2_ are the dimensionality reduction parameters and the dimensionality raising parameters in the fully connected layer, which are, respectively, used to reduce the channel dimension to reduce the amount of calculation and convert to the original channel dimension of u.

c. Finally, the channel weight vector t (1 × 1 × k) and Y (H × W × k) are correspondingly multiplied to achieve the weight calibration of the original feature, and the final output $\tilde{U_{c}}$ is obtained. $\tilde{U_{c}}$ is calculated as follows:

(25) $$\begin{array}{c} \tilde{U_{c}}=f_{\mathrm{scale}}\left(t,Y\right)=t_{c}F_{c} \end{array}$$

Among them, f_*scale*_ is a multiplication function.

#### ReMish

The ReLU function is a widely used activation function in deep learning, and its expression is:


(26) $$\begin{array}{c} ReLU=\left\{ \begin{array}{c} 0,x<0\\ x,x\geq 0 \end{array}\right. \end{array}$$

Compared with sigmoid and tanh, ReLU has the following advantages: for linear functions, ReLU has a stronger expressive ability; for nonlinear functions, the non-negative interval gradient of ReLU is constant, which makes up for the problem of gradient disappearance; ReLU only has a linear relationship, and the calculation is very simple, and ReLU converges much faster than sigmoid and tanh in both forward and backward propagation. Although ReLU can alleviate the disappearance of the gradient and speed up the calculation speed, according to the function definition of ReLU, it can be seen that when the input x < 0, the output is forced to 0, and the gradient is always 0, which can alleviate the problem of overfitting to a certain extent. However, it also caused the calculation results to not converge; the weights could not be updated, resulting in the death of neurons.

The Mish function is a smooth activation function whose expression is:


(27) $$\begin{array}{c} Mish=x*tanh\left(\log\left(1+e^{x}\right)\right) \end{array}$$

Mish positive is unbounded, negative allows some small negative values in an absolutely small area to stabilize the gradient flow of the network, has better generalization ability and the ability to effectively optimize results, allows information to better penetrate the neural network, and improves the learning ability of the network. However, according to the expression, it can be seen that Mish needs logarithms and trigonometry, which adds complexity to the calculation.

**FIGURE 10:**
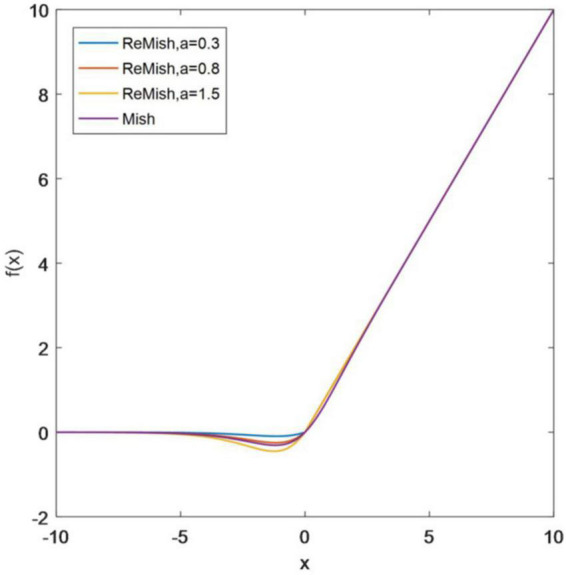
ReMish.

Combining the characteristics of ReLU and Mish, this paper proposes the activation function ReMish, whose shape is shown in Figure 10. The functional form of ReMish is as follows:


(28) $$\begin{array}{c} f\left(x\right)=\left\{ \begin{array}{c} x*tanh\left(\mathrm{alog}\left(1+e^{x}\right)\right),x<0\\ x,x\geq 0 \end{array}\right. \end{array}$$

Among them, a is a random parameter, which changes in real-time according to model training, and finally converges to an appropriate constant.

The improved ReMish function inherits the advantages of both ReLU and Mish functions, mainly including the following two aspects:

(1) The positive semi-axis retains the form of the ReLU function, which can ensure the rapid convergence of the function, alleviate the problem of gradient disappearance, and reduce the computational complexity to a certain extent.
(2) The negative semi-axis inherits the smooth characteristics of the Mish function and nonlinearly corrects the negative semi-axis data, and some small negative values are retained, which solves the problem of ReLU neuron death to a certain extent and stabilizes the gradient flow of the network. In addition, a random parameter a is added, which can effectively control the saturation range of the function.

## Experimental analysis

### Experimental environment and data preparation

Hardware: Processor: Intel(R) Core(TM) I5-8265U CPU @ 1.60 GHz; Graphics card: NVIDIA GeForce MX250; System memory: 8 Gb. Software environment: Cudatoolkit 10.2; PyTorch 1.10; Python 3.8.12; MATLAB R2016A. Operating system: Windows10.

The training of the model requires a large number of citrus disease samples, but obtaining enough disease images is a big challenge. Therefore, we increase the number of samples by expanding the data set before network training, so that the limited data can generate value equivalent to more data, prevent the network from overfitting, and improve the performance of the model. We apply geometric transformations to the image to increase the number: horizontal flipping, vertical flipping, and random cropping. The classification of citrus diseases is not sensitive to the direction of the image, so the processing method of geometric transformation is to simulate the shooting of citrus images from different angles, which is beneficial to restore the real shooting situation.

### Evaluation indicators

This paper studies the classification of citrus diseases, so we choose Accuracy, Precision, Recall, F1-score, and ROC/AUC as indicators to evaluate model performance. Among them, the calculation formulas of Accuracy, Precision, Recall and F1-score are as follows.


(29) $$Accuracy=\frac{TN+TP}{FP+TN+TP+FN}$$

(30) $$Precision=\frac{TP}{TP+FP}$$

(31) $$Recall=\frac{TP}{TP+FN}$$

(32) $$F1-score=\frac{2\left(Precision*Recall\right)}{Precision+Recall}$$

In order to better understand the above formula and the included parameters, first clarify the positive samples and negative samples. Assuming that the images under the citrus canker category are all positive samples, the remaining disease images do not belong to the citrus canker category, that is, they are all negative samples. For the research in this paper, taking the category of citrus canker as an example, TP means that citrus images with canker are correctly predicted to the category “Citrus canker,” and FP means that images of citrus with other diseases are incorrectly predicted to be “citrus canker.” TN means that images of citrus with other diseases are predicted to other disease classes, and FN means that images of citrus with cankers are mispredicted to other disease classes.

Accuracy, Precision, Recall, and F1-score are all commonly used metrics in classification tasks. Accuracy represents the proportion of correctly classified samples to the total number of samples. Precision represents the proportion of the number of correctly classified positive samples to all the predicted positive samples. Recall represents the proportion of the number of correctly classified positive samples to the actual number of positive samples. The F1-score is the harmonic mean of Precision and Recall.

In addition, ROC/AUC are also very important indicators, which are generally represented by a curve graph, namely the ROC curve graph. In the graph, the abscissa and ordinate are FPR and TPR, respectively, the curve is the ROC curve, and the AUC is the area under the ROC curve. The formulas for calculating FPR and TPR are as follows.


(33) $$FPR=\frac{FP}{FP+TN}$$

(34) $$TPR=\frac{TP}{TP+FN}$$

In the above formulas, FPR represents the ratio of the number of misclassified negative samples to the actual number of negative samples, and the meaning of TPR is the same as that of Recall. In practical applications, the closer the ROC curve is to the (0, 1) coordinate, and the closer the area of AUC is to 1, the better the performance of the classifier.

### Experimental design and results

**FIGURE 11:**
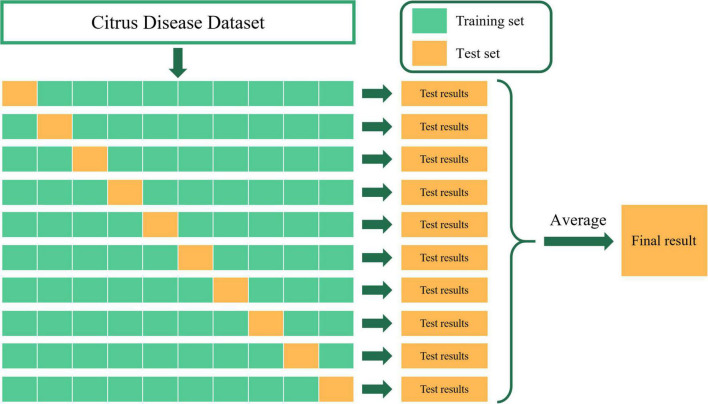
Ten-fold cross-validation.

The experimental dataset in this paper contains five categories of citrus fruits: healthy, citrus canker, citrus black-spot, citrus scab, and citrus anthracnose. We uniformly size the input images to 224 × 224 × 3. After image enhancement and data expansion, a total of 9,258 citrus disease images were obtained. To ensure the reliability and accuracy of our model, we use K-fold cross-validation to train and test the model in all subsequent validation experiments. The basic idea of cross-validation is to group the original data, use most of the samples as the training set to train the model, and use the remaining samples as the test set to test the model, and determine the test error of the small sample, record their sum of squares. Repeat the above process until all samples are predicted and predicted only once. In this paper, we take the value of K as 10, which is 10-fold cross-validation. As shown in Figure 11, we divided the citrus disease data set into 10 groups, and took nine groups as training data and one group as test data in turn, and finally took the average of ten test results as the standard to measure the accuracy of the model. For 10-fold cross-validation, the dataset is randomly divided. So we use 10 times 10-fold cross-validation for training and testing of each model to increase the diversity of dataset division and improve the reliability and accuracy of the model. We retain the best results of each 10-fold cross-validation, and finally take the average of these ten results as the overall classification accuracy of the network model.

In the process of network training, the adjustment of hyperparameters is a critical step. We select the best set of hyperparameters by grid search. Grid search is a very common hyperparameter tuning method. Its essence is to optimize the model by traversing different hyperparameter combinations. When training a network model, the learning rate, batchsize, epoch, and momentum parameters are the first hyperparameters that we need to pay attention to and tune. We choose the learning rate as [0.1, 0.01, 0.001], the batchsize as [16, 32, 64], the epoch as [10, 20, 30], and the momentum parameter as [0.2, 0.4, 0.9]. In order to ensure the reliability of the results in the grid search process, we use 3-fold cross-validation. The resulting optimal hyperparameter combination is: the learning rate is 0.001, the batchsize is 32, the epoch is 20, and the momentum parameter is 0.9. Then there is the choice of the optimizer. We perform grid search again for Adam, RMSprop, and SGDM under the conditions of the above optimal hyperparameter combination, and the Adam optimizer performs the best. In summary, the combination of hyperparameters we finally selected is as shown in Table 4.

**TABLE 4:** Hyperparameter settings.

Hyperparameters	Value
Learning rate	0.001
Epoch	20
Momentum	0.9
Batchsize	32
Optimizer	Adam

It should be noted that in order to ensure the accuracy and reliability of the experiments, all experiments were performed under the same hardware and software environment, and all models used were trained under the same hyperparameter settings.

#### Comparing DS-MENet with other networks

In real life, due to the influence of the internal characteristics of digital photographing equipment and the imaging environment, the captured images are often easily disturbed by some noise, such as the composite noise composed of Gaussian noise and impulse noise (Chen, 2017). Therefore, in order to better simulate the real environment, we randomly select one-third of the samples in the expanded dataset to add noise, that is, compound Gaussian noise and impulse noise and add them to the samples to form a new expanded dataset. We re-tested all models contained in Table 5. For composite noise, we choose the standard deviation of Gaussian noise between 15 and 60 and the ratio of impulse noise between 0.02 and 0.08. Randomly adding different degrees of composite noise to the sample can restore the image to a more realistic shooting quality, so as to verify the anti-interference ability of the model.

**TABLE 5:** Performance of DS-MENet and other network models before and after adding noise to the dataset.

Methods	Accuracy (test set without noise) (%)	Accuracy (test set with added noise) (%)
AlexNet (Krizhevsky et al., 2012)	84.37	79.76
ResNet50 (He et al., 2016)	86.67	82.48
InceptionV4 (Tian et al., 2021)	88.45	86.31
ResNeXt50 (Gp et al., 2020)	89.26	88.56
EfficientNet (Chen et al., 2021)	92.56	91.94
EfficientNetV2 (Sunil et al., 2022)	95.39	94.83
MobileNetV3 (Tarek et al., 2022)	88.64	86.93
DenseNet121 (Huang et al., 2017)	90.06	89.74
DS-MENet	95.25	95.02

For citrus disease classification, in addition to using the DS-MENet model proposed in this paper to test the effect of citrus disease classification, in order to better prove that our model has better reliability and accuracy, we also test with some classical models and state-of-the-art models The experimental results are compared and analyzed, including AlexNet, ResNet50, ResNeXt, InceptionV4, MobileNetV3, EfficientNet, DenseNet121, and EfficientNetV2. AlexNet is a very classic deep convolutional neural network in the early days; ResNet50 proposed a residual block, and the cross-layer connection allows the features of different layers to be transferred to each other, alleviating the problem of gradient disappearance; ResNeXt uses grouped convolution, and also combines the advantages of ResNet and Inception, which has both residual structure and feature layer connection; InceptionV4 uses residual connection to improve InceptionV3, which improves performance; MobileNetV3 combines the advantages of MobileNetV1 and MobileNetV2, and is a more efficient lightweight network; EfficientNetV1 uses neural architecture search technology and introduces a composite coefficient to expand the network from three dimensions of network depth, width and image resolution; EfficientNetV2 introduces an improved progressive learning method based on EfficientNetV1, which has faster training speed and fewer parameters. All in all, the above models have their own characteristics in structure, and also have their own advantages in image classification tasks. So it is necessary to compare our model with them, which can better demonstrate the effectiveness and reliability of our proposed model.

As shown in Table 5, among all models, EfficientNetV2 has the highest average classification accuracy for citrus diseases, this is because EfficientNetV2 uses an improved progressive learning method, that is, the regularization method is dynamically adjusted according to the size of the training image, so as to improve the classification accuracy. The classification effect of our proposed DS-MENet is also good, reaching 95.25%, which is only 0.14% different from EfficientNetV2. After adding random composite noise of Gaussian noise and impulse noise in the dataset, the average classification accuracy of DS-MENet dropped by only 0.23%, and the average accuracy reached the highest on the test set after adding noise. This shows that our model has better anti-interference ability than other models and has higher feasibility in practical scenarios.

#### Evaluation of classification performance of DS-MENet and other networks

**FIGURE 12:**
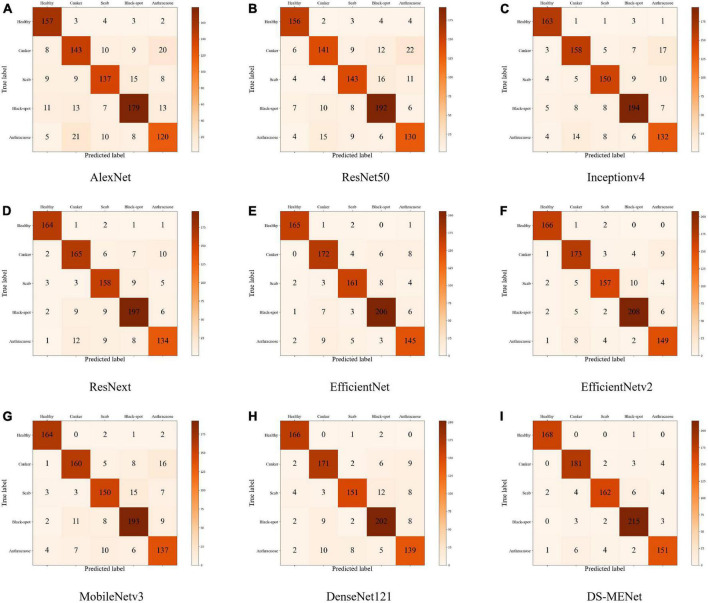
Classification confusion matrix of citrus diseases by different models (the models corresponding to the **(A–I)** confusion matrix are: AlexNet, ResNet50, Inceptionv4, ResNext, EfficientNet, EfficientNetv2, MobileNetv3, DenseNet121, and DS-MENet).

**FIGURE 13:**
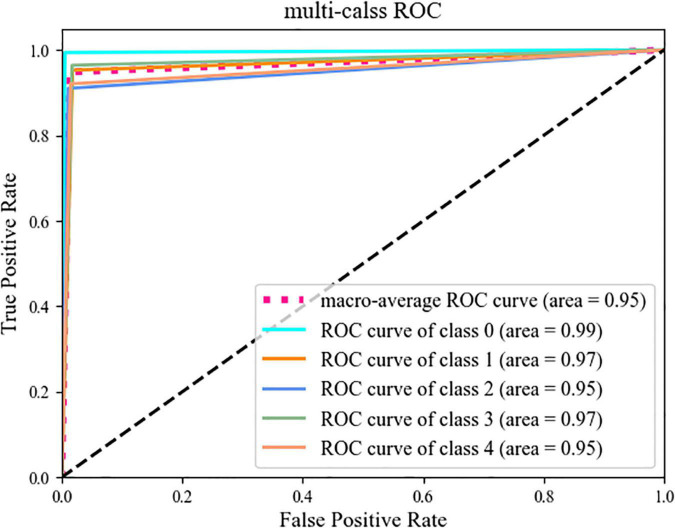
ROC curve.

In this section, we use the expanded dataset after adding noise to demonstrate DS-MENet’s citrus disease classification results and perform model evaluation. As shown in Figure 12, the confusion matrix very intuitively reflects the classification of each type of citrus disease by different models. For the classification performance evaluation of the DS-MENet model, as shown in Table 6, we calculated the average Precision, Recall and F1-score for healthy citrus and four citrus diseases, respectively. The classification effect of healthy citrus on different models is the best, because the surface of healthy citrus has almost no flaws, the image background is simple and the quality is excellent, and the training effect is better. For the classification of the other four citrus diseases, EfficientNetV1, EfficientNetV2, and DS-MENet proposed in this paper have achieved relatively good results, and the average F1-score can basically reach about 90%. However, the classification effect of canker and anthracnose in most models is poor, because the disease characteristics of canker and anthracnose are relatively similar, which increases the difficulty of training and puts forward higher requirements for the ability of the model to extract feature information. Compared with other models, the DS-MENet proposed in this paper still has excellent classification performance, and the average F1-score of healthy citrus, canker, scab, black spot and anthracnose can reach 98.83, 94.27, 93.10, 95.55, and 92.64%. Moreover, our model has a slight improvement in the classification effect of canker and anthracnose, which is because the MCF multi-channel feature fusion structure is added to the last layer of each DenseBlock of DS-MENet. MCF can make full use of the feature information between channels, improve the ability to extract image information, and help to deal with the classification of similar diseases. We also plot the ROC/AUC curve of the DS-MENet model to more intuitively reflect the classification performance. According to Figure 13, it can be seen that the ROC curve is very close to the (0, 1) coordinate, and the area of AUC is also very close to 1, indicating that our proposed DS-MENet model is more suitable for the classification of citrus diseases than some state-of-the-art classification models.

**TABLE 6:** Evaluation of classification performance of DS-MENet and other networks.

Methods	Evaluation indicators	Citrus disease				
		Healthy	Canker	Scab	Black-spot	Anthracnose
AlexNet	Precision (%)	82.63	75.66	81.55	83.64	73.62
	Recall (%)	92.90	75.26	76.97	80.27	73.17
	F1-score (%)	87.46	75.46	79.19	81.92	73.39
ResNet50	Precision (%)	88.14	81.98	79.01	83.49	74.17
	Recall (%)	92.31	74.21	80.34	86.10	79.27
	F1-score (%)	90.18	77.90	79.67	84.77	76.64
Inceptionv4	Precision (%)	90.56	84.95	87.21	88.58	79.04
	Recall (%)	96.45	83.16	84.27	87.00	80.49
	F1-score (%)	93.41	84.05	85.71	87.78	79.76
ResNeXt50	Precision (%)	95.35	86.84	85.87	88.74	85.90
	Recall (%)	97.04	86.84	88.76	88.34	81.71
	F1-score (%)	96.19	86.84	87.29	88.54	83.75
EfficientNet	Precision (%)	97.06	89.58	92.00	92.38	88.41
	Recall (%)	97.63	90.53	90.45	92.38	88.41
	F1-score (%)	97.34	90.05	91.22	92.38	88.41
EfficientNetv2	Precision (%)	96.51	90.10	93.45	92.86	88.69
	Recall (%)	98.22	91.05	88.20	93.27	90.85
	F1-score (%)	97.36	90.57	90.75	93.06	89.76
MobileNetv3	Precision (%)	94.25	88.40	85.71	86.55	80.12
	Recall (%)	97.04	84.21	84.27	86.55	83.54
	F1-score (%)	95.62	86.25	84.98	86.55	81.79
DenseNet121	Precision (%)	94.32	88.6	92.07	88.99	84.76
	Recall (%)	98.22	90.00	84.83	90.58	84.76
	F1-score (%)	96.23	89.29	88.30	89.78	84.76
DS-MENet	Precision (%)	98.25	93.30	95.29	94.71	93.21
	Recall (%)	99.41	95.26	91.01	96.41	92.07
	F1-score (%)	98.83	94.27	93.10	95.55	92.64

#### Verifying the effectiveness of data preprocessing

In order to more comprehensively verify the validity of the citrus disease dataset after image preprocessing (image enhancement and data expansion), we performed the same data expansion on the dataset without image enhancement and enhanced by the HFLF-MS algorithm, respectively. Methods include horizontal flipping, vertical flipping, random cropping. Finally we got the original dataset without image enhancement, the original dataset with HFLF-MS image enhancement, the extended dataset without image enhancement and the extended dataset with HFLF-MS image enhancement. Afterward, we feed these datasets into the DS-MENet model proposed in this paper for training and testing.

As can be seen from Table 7, compared with the original dataset, whether image enhancement is performed or not, the data expansion can improve the accuracy. This is because the sample size of the original dataset is smaller, and the data expansion can Provide more samples for the model, increase the diversity of samples, and improve the generalization ability of the model.

**TABLE 7:** Classification accuracy of different networks before and after image augmentation and dataset augmentation.

Image enhancement methods	Original dataset (%)	Extended dataset (%)
No image enhancement	83.17	89.62
HFLF-MS	85.94	95.25

#### Verifying the effectiveness of the improved activation function ReMish

**FIGURE 14:**
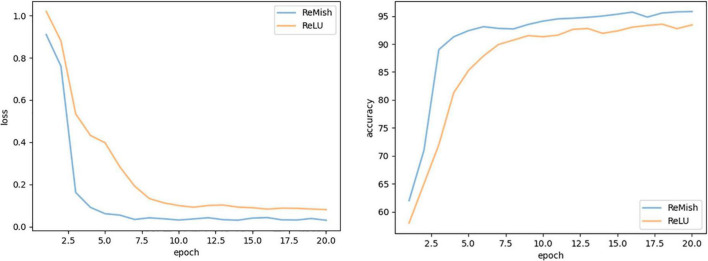
Performance comparison of ReLU and ReMish.

In this section, we use the expanded dataset after adding noise to conduct experiments to verify that the activation function ReMish can improve the expression ability of the neural network to the model. We conducted a comparative experiment on the network model before and after the improved ReLU activation function. The experimental model uses the DS-MENet proposed in this paper. Figure 14 shows the Accuracy and Loss training curves before and after the activation function is improved. Compared with the ReLU activation function, the ReMish activation function can improve the convergence speed of the network. As shown in Table 8, the classification accuracy of citrus disease images is also improved by 1.6%.

**TABLE 8:** Verifying the effectiveness of the improved activation function ReMish.

Activation function type	Accuracy (%)	Loss
ReLU	93.42	0.06
ReMish	95.02	0.03

#### Verifying the effectiveness of depthwise separable convolution

In this section, we use the expanded dataset after adding noise to verify that the depthwise separable convolution can reduce network parameters and thus reduce the amount of computation. We design different network models. As can be seen from Table 9, among all network models, our proposed DS-MENet has a relatively short training time. Compared with ResNet and DenseNet121, the training time of ResNet-DS and DenseNet121-DS is reduced, but this is at the cost of accuracy, and adding depthwise separable convolution will inevitably bring about a drop in accuracy.

**TABLE 9:** Verifying the effectiveness of depthwise separable convolutions.

Normal networks	ResNet50	DenseNet121	DenseNet121-DS	ResNet50-DS	DS-MENet
Training time (s)	1 h 37 m 34 s	2 h 20 m 56 s	2 h 4 m 12 s	1 h 22 m 24 s	2 h 18 m 29 s
Accuracy (%)	82.48	89.74	88.86	80.65	95.02

#### Verifying the effectiveness of the MCF backbone enhancement method

In this section, we use the expanded dataset after adding noise to verify the effectiveness of the MCF method. We design different network structures. As can be seen from Table 10, after adding MCF to ResNet and DenseNet, the accuracy has been slightly improved. Among them, the classification accuracy of DenseNet121-MCF and DS-MENet can reach more than 90%. This is because MCF enhances the backbone of DenseBlock, which can enhance the utilization of feature maps by fusing grouped convolutions with feature maps between adjacent groups, thereby maximizing the extraction of detailed features of citrus diseases during operation. But it can also be clearly seen that the accuracy improvement brought by the addition of MCF comes at the expense of increasing training time.

**TABLE 10:** Verifying the effectiveness of the MCF backbone enhancement method.

Normal networks	ResNet50	DenseNet121	DenseNet121-MCF	ResNet50-MCF	DS-MENet
Training Time	1 h 37 m 34 s	2 h 20 m 56 s	2 h 43 m 15 s	1 h 56 m 44 s	2 h 18 m 29 s
Accuracy (%)	82.48	89.74	94.31	87.55	95.02

#### Ablation experiment

To verify the effectiveness of the method proposed in this paper, we conduct ablation experiments. In the ablation experiments, we use the same experimental environment, using a 10-fold cross-validation approach, using the expanded dataset after adding noise to some samples. We use DenseNet121, InceptionV4, and ResNeXt as the overall structure of the network, plus one or more of the methods proposed in this paper to form ablation experiments. In this experiment, we want to verify the effectiveness of depthwise separable convolution, MCF, ReMish, respectively. The experimental results are shown in the Table 11.

**TABLE 11:** Ablation experiment results.

Module	DenseNet121		InceptionV4		ResNeXt	
	Accuracy (%)	Training time	Accuracy (%)	Training time	Accuracy (%)	Training time
Baseline	89.74	2 h 20 m 56 s	86.31	1 h 58 m 31 s	88.56	2 h 15 m 27 s
DS	88.86	2 h 4 m 12 s	84.16	1 h 40 m12 s	87.12	1 h 54 m 42 s
MCF	94.31	2 h 43 m 15 s	89.97	2 h 28 m 43 s	92.05	2 h 48 m 29 s
ReMish	90.33	2 h 10 m 09 s	87.81	1 h 42 m 51 s	89.66	2 h 03 m 04 s
DS+ReMish	89.18	1 h 51 m 31 s	86.44	1 h 23 m 03 s	88.45	1 h 44 m 35 s
DS+MCF	93.42	2 h 27 m 18 s	88.14	2 h 04 m 15 s	91.17	2 h 28 m 22 s
MCF+ReMish	95.97	2 h 33 m 02 s	91.21	2 h 13 m 55 s	93.18	2 h 31 m 05 s
DS+ReMish+MCF	95.02	2 h 18 m 29 s	90.03	1 h 56 m 28 s	91.83	2 h 16 m 08 s

First, it can be seen from the Table 11 that depthwise separable convolution, MCF and ReMish all contribute to the improvement of citrus classification accuracy, among which DS-MENet has the highest average classification accuracy of 95.02%. When only one method is added to DenseNet121, MCF has the highest contribution to classification accuracy, and depthwise separable convolution has the highest contribution to training time. For the improvement of accuracy, using MCF to process DenseBlock can improve the utilization of feature information between channels and enhance the ability of the network to extract detailed features of citrus diseases. For the shortening of training time, depthwise separable convolution can reduce the amount of computation and parameters, thereby improving computational efficiency. When DenseNet121 adds two methods, the combination of MCF+ReMish achieves the highest classification accuracy, and the combination of DS+ReMish minimizes the training time. The ReMish activation function can not only make the model converge faster and reduce the training time, but also make some contributions to the expressiveness of the data and the improvement of the accuracy. When three methods are added to DenseNet121, the DS-MENet proposed in this paper is formed. The average classification accuracy rate can reach 95.02%, and the training time is 2 h 18 m 29 s. Compared with DenseNet121-MCF-ReMish, although the accuracy is reduced by 0.95%, the gap is small. Compared with DenseNet121-DS-ReMish, although the training time has increased a little, the classification accuracy has been significantly improved. Besides validating DS, ReMish and MCF on DenseNet121, we also conduct ablation experiments in InceptionV4 and ResNeXt, respectively. As can be seen from the table, adding three structures on InceptionV4 and ResNext can also achieve good classification performance. However, compared with DenseNet121 as the backbone network, there is still a slight gap between InceptionV4 and ResNeXt. In the research of this paper, the biggest difficulty is the accurate classification of similar diseases, which requires high retention and extraction of detailed information. DenseNet121 can make full use of shallow and deep information through the connection between layers, maximize the use of detailed information, and be more conducive to the classification of similar diseases. Therefore, it is a better choice to use DenseNet121 as the backbone network. A comprehensive analysis of all the ablation experimental results shows that DS-MENet can achieve a balance between training time and classification accuracy, and has relatively good classification performance and use value.

#### Supplementary experiment

To better verify the performance of DS-MENet, we train and test again on PlantVillage, Stanford cars and ImageNetDogs. Table 12 provides details about the dataset, including the number of classes, total number of samples, and sources.

**TABLE 12:** Public dataset details.

Dataset	Category	Total	Available
PlantVillage	38	55,400	https://www.kaggle.com/datasets/hiyash99/plantvillage
Stanford cars	196	16,185	https://www.kaggle.com/datasets/jutrera/stanford-car-dataset-by-classes-folder
ImageNetDogs	120	20,580	http://vision.stanford.edu/aditya86/ImageNetDogs/

We use three public datasets to test the model proposed in this paper, and the test results are shown in the Table 13. It can be seen that our model has good performance on three public datasets, indicating that our proposed model has good generalization ability on other datasets.

**TABLE 13:** Average classification accuracy of DS-MENet on public datasets.

Dataset	Accuracy (%)
PlantVillage	96.16
Stanford cars	95.41
ImageNetDogs	94.32

## Conclusion

This paper proposes a method for image recognition and classification of citrus diseases based on HFLF-MS and DS-MENet models. Firstly, the HFLF-MS algorithm is used to enhance the citrus disease image to highlight the details of the image and improve the quality of the image. Then, the enhanced images are preprocessed by horizontal flipping, vertical flipping, random cropping, and other methods to expand the number of datasets. The expanded dataset is then fed into the DS-MENet network model. DS-MENet adopts DenseNet-121 as the basic network structure and enhances the backbone of Dense Block by introducing depthwise separable convolution and MCF algorithm based on grouped convolution, multi-channel fusion, and SE attention mechanism to improve the network’s ability to extract feature information of citrus disease images. The experimental results show that using the 10-fold cross-validation method, DS-MENet can achieve an average classification accuracy of 95.02% on the dataset with random compound noise added. This shows that the citrus disease classification method based on the combination of HFLF-MS and DS-MENet network has good anti-interference ability and has certain feasibility in real life.

For the surface disease defects of citrus and other fruits, image recognition and classification technology is still an important research direction, which is of great significance to ensure the quality of fruits and improve the development of the agricultural economy. Even though deep learning has achieved outstanding performance in the identification and classification of various fruit diseases, it still needs to be further improved. The citrus disease samples collected in this paper are not enough, and it is necessary to further expand the data set and test scale, so as to improve the generalization ability of the model and better meet the needs of agricultural development.

## Data availability statement

The original contributions presented in this study are included in the article/supplementary material, further inquiries can be directed to the corresponding author.

## Author contributions

XL: conceptualization, data curation, and writing—original draft. JZ and YHH: formal analysis and resources. GZ: funding acquisition. YWH: investigation and software. XL and WC: methodology. MH: project administration, supervision, and validation. LL: visualization and writing—review and editing. All authors contributed to the article and approved the submitted version.

## Conflict of interest

The authors declare that the research was conducted in the absence of any commercial or financial relationships that could be construed as a potential conflict of interest.

## Publisher’s note

All claims expressed in this article are solely those of the authors and do not necessarily represent those of their affiliated organizations, or those of the publisher, the editors and the reviewers. Any product that may be evaluated in this article, or claim that may be made by its manufacturer, is not guaranteed or endorsed by the publisher.
